# Characterisation and phylogenetic analysis of the complete mitochondrial genome of two endemic lampreys from Greece (*Caspiomyzon hellenicus* and *Caspiomyzon graecus*) using Long-Read technology

**DOI:** 10.1007/s11033-025-10476-5

**Published:** 2025-04-05

**Authors:** Chrysoula Gubili, Orezia Seitidou, Romina Batista, Paraskevi Papadopoulou, Aris Christidis, Stelios Triantafillidis, Argyrios Sapounidis

**Affiliations:** 1 Fisheries Research Institute, Hellenic Agricultural Organisation-DIMITRA, Nea Peramos, Kavala, 64007 Greece; 2 School of Science, Engineering, and Environment, University of Salford, Salford, M5 4WT UK; 3 Department of Biological and Environmental Sciences, University of Gothenburg, Box 463, Gothenburg, SE-405 30 Sweden

**Keywords:** Petromyzontidae, Greece, Mitogenome, Phylogeny, Next generation sequencing, Long-reads

## Abstract

**Background:**

The genus *Caspiomyzon* is comprised of three species, two of which are found in Greece (*Caspiomyzon hellenicus* and *Caspiomyzon graecus*). Both species are endemic with very restricted distribution and are classified as Critically Endangered. *Caspiomyzon hellenicus* is restricted to Tenagi, Philippi and *C. graecus* to Louros River. No studies have characterized their mitochondrial genomes.

**Methods and results:**

The complete mitochondrial genome of *C. hellenicus* and *C. graecus* was generated with Oxford Nanopore long-read technology, and it was processed using various bioinformatics approaches. The final assembled contig length was 16,763 bp and 17,123 bp, respectively, and composed of 13 protein-coding genes, two ribosomal RNA genes, 22 tRNA genes, and two control regions. Repetitive sequences were detected between and within the control regions. The overall GC composition was approximately 36% for both species. The results of phylogenetics analysis using Bayesian inference and Maximum likelihood methods revealed that the *Geotria australis* species was sister to northern hemisphere lampreys, whereas *Mordacia* species constitutes a monophyletic group. Divergence time between the Greek *Caspiomyzon* species took place at approximately 0.7 Mya.

**Conclusions:**

This study enhanced our understanding of the taxonomic and phylogenetic relationships within the *Caspiomyzon* genus from Greece based on the characterization of the full mitochondrial genomes from long-reads technology. Such efforts can aid their conservation and management locally.

## Introduction

European freshwater fishes is the second richest group amongst European vertebrates, and the second most threatened in Europe [1]. Freshwater fish species comprise 40% of all fish diversity and 25% of all vertebrates [2] and provide several ecosystem services [3]. Greece has a diverse ichthyofauna with more than 177 freshwater fishes, and the highest fish endemism in the Mediterranean region [4]. There are 47 endemic species (26.55%, 138 native species) and 39 that have been introduced by humans [4, 5]. However, anthropogenic impacts can affect environmental characteristics [6], leading to losses of freshwater habitat availability, and threaten biodiversity. Despite the importance of freshwater fauna as a national heritage in Greece [5], its high species richness and role in ecosystems have received limited attention in comparison to marine fishes [7]. This is of extreme importance, particularly as evidence suggests that freshwater species are more sensitive to environmental changes than marine species [8].

**Fig. 1:**
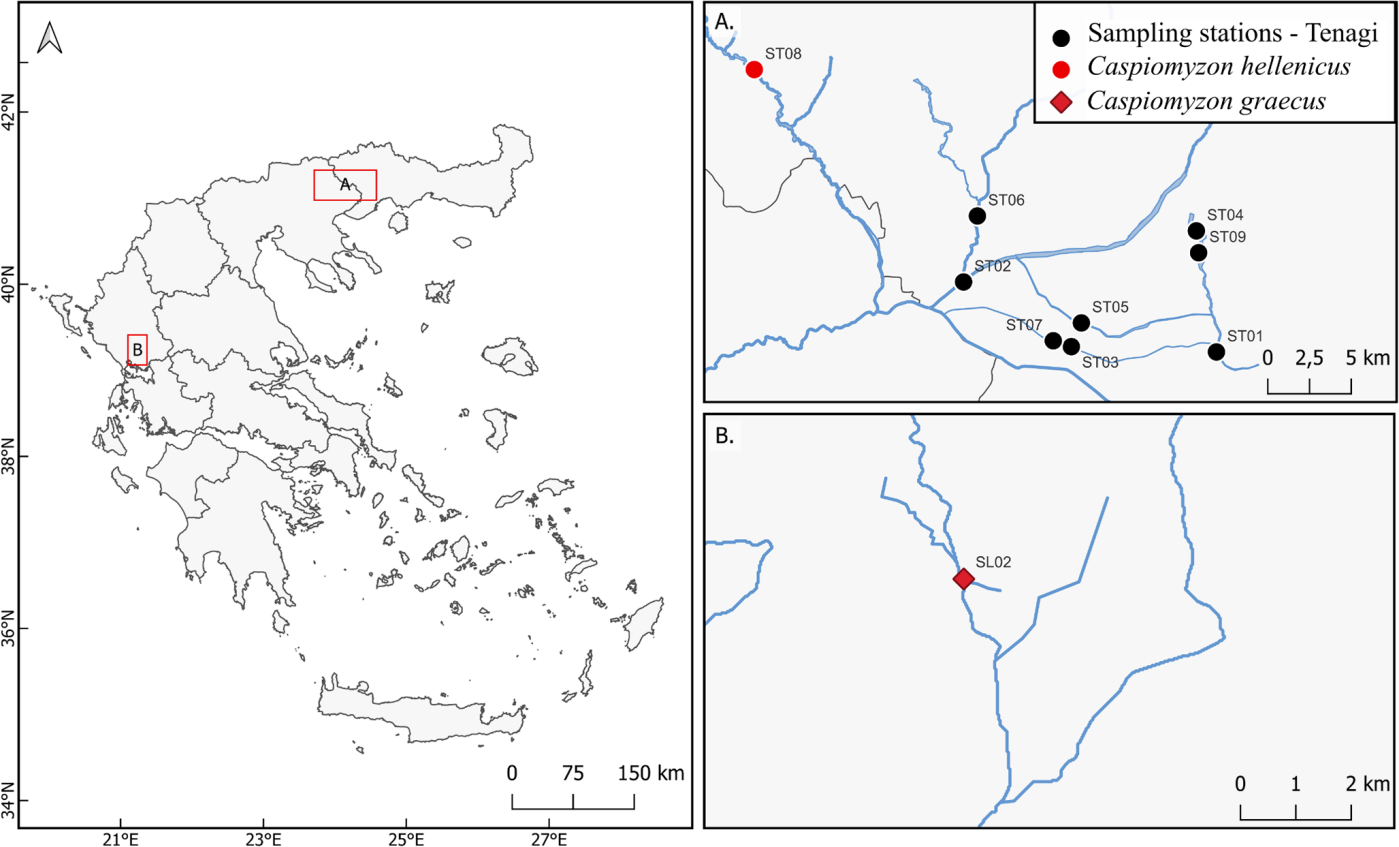
Approximate sampling locations using electrofishing at **A** Tenagi, Philippi and **B** Louros River

The area of Tenagi (Philippi, Eastern Macedonia, Greece; Fig. 1A) is approximately 88 km^2^ and it hosts approximately 12.5% of the Greek freshwater fishes [9], two of which are endemic [*Caspiomyzon hellenicus* (Vladykov, Renaud, Kott & Economidis, 1982); *Cobitis punctilineata* Economidis & Nalbant, 1996]. It consists of a complex of small rivers and streams (e.g., Aggitis River, Doxatos stream, Voirani, Agia Varvara, and Kefalari Springs) and the deepest peat deposit in the world with a depth of 198 m [10], making it a very productive agricultural landscape. However, the continuous and intensive agricultural activities have significantly affected the biochemical characteristics of the area, that could lead to mass death of fishes and local extirpation of species [11].

Among the freshwater species that are currently under threat are the lampreys [6], with 15.56% of species worldwide currently listed as threatened (Vulnerable, Endangered, and Critically Endangered) under the International Union for Conservation of Nature (IUCN) Red List. All lampreys have complex life cycles that include an extended larval stage that lasts up to seven years, a brief transformative period of one to two months, and a short adult stage of several months to two years [12]. Moreover, they have developed multiple life history strategies including both anadromous and resident life histories, which become more variable as they also possess both parasitic and non-parasitic forms [13]. They also have an anti-tropical distribution with two families being endemic to the southern hemisphere whereas the third, which contains the highest number out of the currently over 50 recognised species, is solely found in the northern hemisphere [14]. Therefore, the evolutionary reconstruction and species delimitation of lampreys become notably challenging, and are exacerbated by the morphological variability within species.

*Caspiomyzon* is a genus in the family Petromyzontidae of the northern hemisphere with a very restricted geographic distribution. There are only three species currently recognized within the genus, two of which [*C*. *hellenicus* and *Caspiomyzon graecus* (Renaud & Economidis, 2010)] inhabit isolated freshwater streams of Greece (non-parasitic forms), and one [*Caspiomyzon wagneri* (Kessler, 1870)] that is restricted to the Caspian Sea basin (parasitic form). The Macedonia Greek brook lamprey (*C*. *hellenicus*) is classified as Critically Endangered by the Greek Red Book of threatened fauna and by the IUCN Redlist of threatened species [15]. It is also included in Annex II of both the Habitats Directive (Directive 92/43/EEC) and the Bern Convention (Bern Convention 1979). Additionally, this lamprey is endemic to the wider area of Tenagi, Philippi, and is found in Aggitis River, and in the Ai Giannis, Kefalari and Mylopotamos tributaries [5, 15]. Its congeneric species, the Epirus brook lamprey (*C*. *graecus*) from which it was separated ca. 0.35 Mya [16], has been recently identified taxonomically and has been recently assessed as Critically Endangered [17]. Similarly, it exhibits an equally narrow geographic distribution, which is restricted to the Louros River and one of its tributaries, Filippias [18]. Both species were previously included in the *Eudontomyzon* genus and were moved to the genus *Caspiomyzon* following taxonomic revision [19].

Third generation sequencing technology (TGS) and long-reads sequencing data allow the *de novo* genome assembly of non-model organisms [20], the study of animal molecular systematics and evolution, as well as the identification of various species [21]. Oxford Nanopore Technologies (ONT) offer a relatively cost-effective approach in sequencing; for example, the whole mitochondrial genome can be generated from a single read, overcoming potential overlap, uncertainties of short-read and Sanger sequencing approaches [22]. Fish mitogenomes have small sizes that vary in length (15–20 Kb), simple structure, high evolution rate, and low level of recombination [23–24], making them very effective and reliable molecular markers in reconstructing phylogenies.

This study will attempt to resolve the phylogenetic relationship of two threatened Greek species with the application of molecular tools. We sequenced, assembled, and annotated the mitochondrial genomes of both *Caspiomyzon* species in Greece using long-read sequencing technology of ONT. Additionally, we examined their phylogenetic placement among extant lamprey species using complete mitogenomes from public databases (GenBank).

## Materials and methods

### Sample collection and species identification

One *Caspiomyzon hellenicus* individual and one *Caspiomyzon graecus* specimen were collected from Aggitis River in September 2023 and from Louros River in June 2023, respectively (Fig. 1; Table 1). Additionally, individuals from the most recorded freshwater fishes (eight species) of the Tenagi area (Philippi) were collected between August to September 2023 (Fig. 1; Table 1). All necessary field permits were obtained (Permit Numbers: 20980/28-04-2023 and 37899/15-03-2023).

**Table 1:** Species list of fish found currently and historically in the area Tenagi, Philippi. **#** species from Louros river, Epirus.

Species	Family	Status IUCN	Accession number
*Alburnoides strymonicus*	Leuciscidae	Not Evaluated	This study
*Alburnus alburnus*	Leuciscidae	Least Concerned	AB239593
*Barbus strumicae*	Cyprinidae	Least Concerned	This study
*Carassius gibelio*	Cyprinidae	Least Concerned	KU896991
*Caspiomyzon hellenicus*	Petromyzontidae	**Critically Endangered**	This study
*Caspiomyzon graecus#*	Petromyzontidae	**Critically Endangered**	This study
*Chondrostoma vardarense*	Leuciscidae	Not Threatened	This study
*Cobitis punctilineata*	Cobitidae	**Vulnerable**	This study
*Economidichthys pygmaeus*	Gobiidae	Least Concerned	This study
*Gambusia holbrooki*	Poeciliidae	Least Concerned	OP882646
*Gasterosteus aculeatus*	Gasterosteidae	Least Concerned	NC_041244
*Lepomis gibbosus*	Centrarchidae	Least Concerned	MF621726
*Leucaspius delineatus*	Leuciscidae	Least Concerned	NC_020357
*Oxynoemacheilus bureschi*	Nemacheilidae	Least Concerned	This study
*Petroleuciscus borysthenicus*	Leuciscidae	Least Concerned	This study
*Phoxinus strymonicus*	Leuciscidae	**Endangered**	This study
*Pseudorasbora parva*	Gobionidae	Least Concerned	NC_015614
*Rhodeus amarus*	Acheilognathidae	Least Concerned	NC_031538
*Rutilus rutilus*	Leuciscidae	Least Concerned	PP928771
*Squalius orpheus*	Leuciscidae	Least Concerned	Not generated
*Tinca tinca*	Tincidae	Least Concerned	NC_008648
*Vimba melanops*	Leuciscidae	Data Deficient	NC_031539

Specimens were collected using the portable electrofishing device Hans Grassl ELT60IIHI (300 V and 60 Hz), according to the EU CEN 2003 protocol. This sampling method is very efficient for recording ichthyofauna in rivers without harming fishes (Directives 92/43 and 2000/60). Fishes were anesthetized by the closed circuit of the electrofishing device and subsequently identified to species level [5, 25]. Fin clips from the anesthetized fish were collected and preserved in absolute ethanol until further processing. All fish were subsequently released back into their original habitat.

### DNA extraction, nanopore library preparation, and sequencing

Genomic DNA was extracted from fins using the MagAttract HMW DNA Kit (Qiagen, Germany) according to the manufacturer’s protocol. DNA was assessed for quality and quantity using a Qubit4 fluorometer with a Qubit dsDNA HS Assay kit (Invitrogen, ThermoFisher). The extraction with the highest quality DNA was selected for further use.

Nanopore libraries were prepared with 600 ng of genomic DNA from each species following the protocol for the native barcoding kit 96 V14 (SQK-NBD114.96, Oxford Nanopore Technologies). Briefly, DNA repair and end-prep was carried out by adding 0.875 µl NEBNext FFPE DNA Repair Buffer, 0.875 µl Ultra II End-prep reaction buffer, 0.75 µl Ultra II End-prep enzyme mix, 0.5 µl NEBNext FFPE DNA Repair Mix (New England Biolabs) per sample. The repaired/end-prepped DNA was subsequently incubated at 20 ºC for 5 min and 65 ºC for 5 min. The end repaired genomic DNA was ligated to Nanopore barcodes by adding 0.75 µl of end-prepped DNA to 3 µl of nuclease-free water, 1.25 µl Nanopore barcodes to each sample, and 5 µl Blunt/TA Master Mix (New England Biolabs). Ligations were carried out at room temperature for 20 min. Barcoded genomic DNA were purified using 0.4X magnetic beads (AMPure XP, Beckman Coulter), pooled and ligated to the sequencing adaptors by adding 5 µl Native Adapter (NA), 10 µl NEBNext Quick Ligation Reaction Buffer (5X), and 5 µl Quick T4 DNA Ligase to 30 µl of pooled barcoded sample. Libraries were purified by 20 µl magnetic beads and eluted in 25 µl elution buffer (Oxford Nanopore Technologies). The sequencing flow cell was primed using 1,000 µl of priming mix (Flush Tether and Flush buffer, ONT). After priming, the 32 µl library was mixed with 100 µl Sequencing Buffer (SB) and 68 µl Library Beads (LIB). The final library was loaded onto a Nanopore FLO-PRO114M (R10.4.1) flow cell and sequenced on a PromethION P2 Solo for 24 h (Oxford Nanopore Technologies). Real-time basecalling was performed in MinKnow 23.11.3 using the high-accuracy model to produce fastq files.

### Mitogenome assembly and annotation of *Caspiomyzon* species from Greece

The complete mitogenome of *Petromyzon marinus* was obtained from GenBank (Table 2) and used on Minimap 2.22 [26] as reference for the draft assembly with the option *-ax map-ont*. Each assembly was polished using the alignment information of their corresponding raw reads by Minimap and Racon 1.5.0 [27]. Polishing was performed three times to improve sequence accuracy. Both reconstructed mitogenomes were annotated on MitoFish 4.07 [28] and confirmed on Geneious Prime 2024.4 (http://www.geneious.com). The consensus was inspected, and polymorphic sites were carefully checked and adjusted against mitogenomes of the family as references to ensure the integrity of reading frames of the protein-coding genes and the expected secondary structure of tRNAs and rRNAs. Predicted gene boundaries were manually adjusted and the annotated mitogenome was visualised in MitoFish and edited on Inkscape 1.3 (https://inkscape.org/).

**Table 2:** Lampreys used for phylogenetic analyses. * species used as a reference genome for the annotation of both *Caspiomyzon* species.

A/A	SPECIES	Accession Number	Mitogenome size (bp)
1	*Entosphenus lethophagus*	NC_066901	16,157
2	*Entosphenus minimus*	NC_066903	16,206
3	*Entosphenus similis*	NC_066904	16,174
4	*Entosphenus tridentatus*	NC_066902	16,151
5	*Eudontomyzon morii*	NC_025582	16,172
6	*Geotria australis*	OP781304	17,094
7	*Ichthyomyzon fossor*	NC_025552	16,150
8	*Ichthyomyzon gagei*	KY056640	16,359
9	*Ichthyomyzon unicuspis*	NC_025553	16,163
10	*Lampetra aepyptera*	NC_026917	16,236
11	*Lampetra appendix*	NC_025583	16,169
12	*Lampetra fluviatilis*	Y18683	16,159
13	*Lampetra richardsoni*	NC_066900	16,120
14	*Lethenteron camtschaticum*	KF701113	16,272
15	*Lethenteron reissneri*	AB565771	16,461
16	*Mordacia mordax*	OP781305	17,233
17	*Mordacia praecox*	OP781306	17,065
18	Petromyzon marinus*	MW856857	16,067

The nucleotide composition was analysed with MEGA 11.0.13 [29]. Strand skew values were calculated according to [30]: AT skew = (A - T)/(A + T) and GC skew = (G - C)/(G + C), where A, T, C, G are the four bases.

### Phylogenetic analyses and divergence time estimates among the Petromyzontiadae family

Full mitochondrial genomes of 18 Petromyzontidae species were obtained from GenBank (Table 2). Datasets were aligned in MEGA using ClustalW and the default parameters. The aligned sequences were trimmed manually to remove unaligned codons and nucleotides. To infer the relationships among Petromyzontidae, we estimated Maximum Likelihood (ML) phylogenies using IQ-TREE 2.3.4 with 1,000 bootstraps replicates [31]. Bayesian inference analysis (BI) was also carried out with MrBayes v.3.2.7a [32] following the selection of the most appropriate model of evolution as estimated on IQ-TREE for each data set. Analyses were performed for two Markov chains of 1,000,000 generations each with sampling every 100 generations resulting in 10,000 trees, of which the first 25% were discarded as burn-in. Topologies were visualised using Figtree 1.4.4 (http://tree.bio.ed.ac.uk/software/figtree/).

Additionally, the divergence times of the family was estimated using BEAST 2.7.7 [33] following [16]. Briefly, the bModelTest module [34] was used to select the best model during the *mcmc* run, instead of setting substitution models for each partition. Additionally, a lognormal relaxed clock was assumed and a rate of 0.01 substitutions/site/MY was applied. A Yule speciation prior was used for all partitions and analysis was run for 10^8^ generations, and logging parameters every 10,000 generations. A burn-in of 10% was applied and convergence of analysis was checked on Tracer 1.7.2 [35] by inspecting Effective Sample Size (ESS) values. The mitogenomes of *Eptatretus burgeri* (Accession Number NC_002807) and *Myxine glutinosa* (Accession Number NC_002639) were used as an outgroup.

## Results

### Mitogenome characterization, annotation, and sequence analysis

**Fig. 2:**
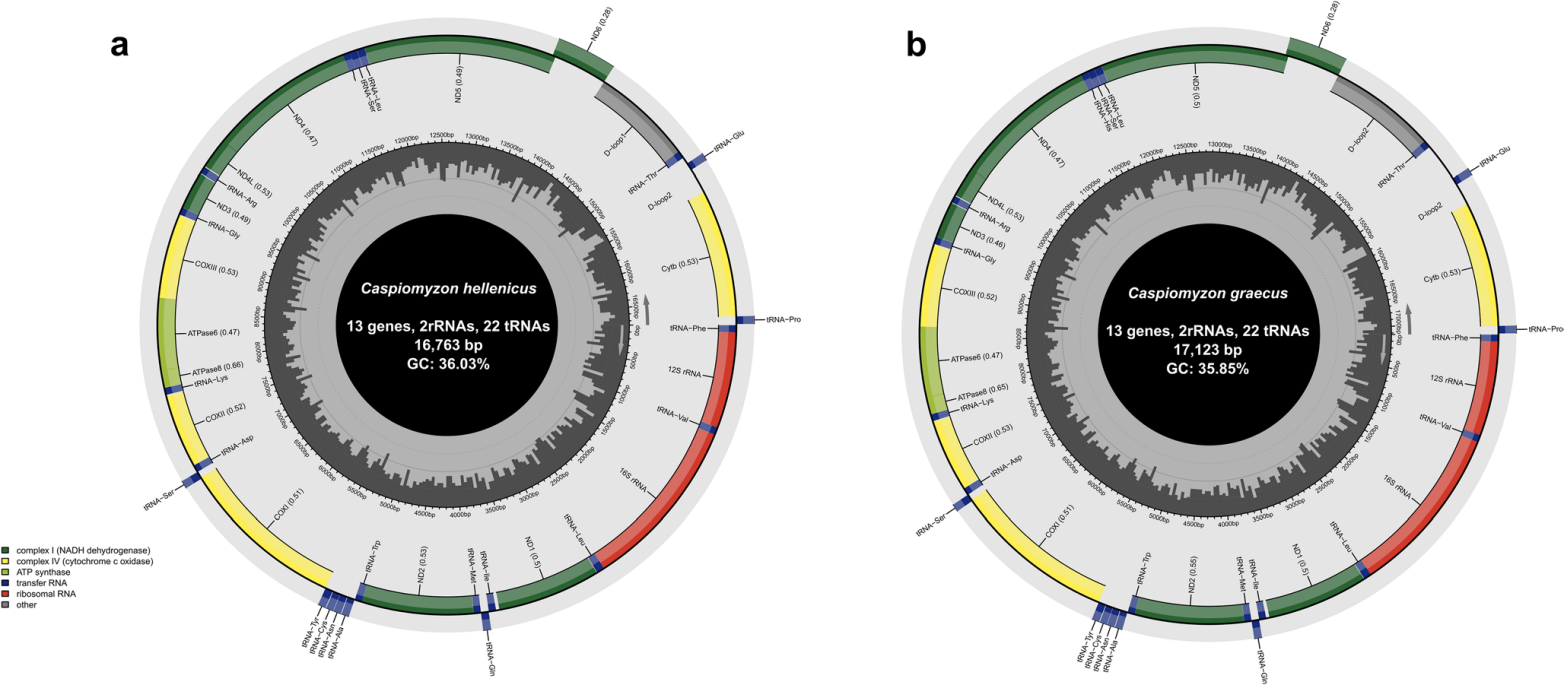
Circular DNA mitochondrial genome map of **a***Caspiomyzon hellenicus* and **b***Caspiomyzon graecus*. The annotated map depicts 22 transfer RNA (tRNA) genes, 13 protein-coding genes (PCGs), two ribosomal RNA genes (rrnS: 12S ribosomal RNA and rrnL: 16S ribosomal RNA), and two putative D-loop regions.

A total of 556,000 and 100,287 reads were generated for *C. hellenicus* and *C. graecus*, respectively. The *de novo* assembly produced single circular contigs of 16,763 and 17,123 bp in length for *C. hellenicus* and *C. graecus* (Accession numbers: PQ845996-PQ845995) respectively. The GC content was 36.03% for *C. hellenicus* and 35.85% for *C. graecus* (Fig. 2). The MitoFish annotation pipeline identified 13 protein-coding genes (PCGs), 22 transfer RNAs (tRNA), two ribosomal RNAs (rRNA), two non-coding control regions, and several intergenic regions (of different length) in both mitogenomes, which were consistent with those reported from other lamprey species. The *C. hellenicus* mitochondrial genome has a positive AT skew (0.033) and a negative CG skew (0.287) with base frequencies of A = 33.05%, C = 23.19%, G = 12.84%, and T = 30.91%. The positive strand contained all PCGs except for *nd6*, two rRNAs and 14 tRNAs genes, whereas the remaining eight tRNAs were located on the negative strand (Fig. 2a). Similarly, the *C. graecus* mitogenome has a positive AT skew (0.032) and a negative CG skew (0.285) with base frequencies of A = 33.11%, C = 23.02%, G = 12.82%, and T = 31.05%. The positive strand contained 12 PCGs, two rRNAs, and 14 tRNAs, whilst the negative strand contained one PCG (*nad6*), eight tRNAs (Fig. 2b). All PCG for both species started with ATG, except for COXI, which has GTG as its start codon (Table 3).

**Table 3:** Organisation of the mitogenomes of two Greek lamprey species (*Caspiomyzon Hellenicus* and *Caspiomyon graecus*).

Genes	Caspiomyzon hellenicus	Genome size (bp)	Caspiomyzon graecus	Genome size (bp)	Start Codon	End Codon	Strand
tRNA-Phe	1–67	67	1–67	67			**+**
12 S rRNA	68–966	899	68–968	901			**+**
tRNA-Val	967–1037	71	969–1039	71			**+**
16 S rRNA	1038–2659	1622	1040–2662	1623			**+**
tRNA-Leu	2660–2733	74	2663–2736	74			**+**
ND1	2738–3703	966	2741–3706	966	ATG	TAA	**+**
tRNA-Ile	3735–3802	68	3738–3805	68			**+**
tRNA-Gln	3805–3875	71	3808–3878	71			**-**
tRNA-Met	3876–3943	68	3879–3946	68			**+**
ND2	3945–4988	1044	3948–4991	1044	ATG	TAG	**+**
tRNA-Trp	4987–5054	68	4990–5057	68			**+**
tRNA-Ala	5057–5124	68	5060–5127	68			**-**
tRNA-Asn	5130–5198	69	5133–5201	69			**-**
tRNA-Cys	5201–5267	67	5204–5270	67			**-**
tRNA-Tyr	5273–5343	71	5276–5345	70			**-**
COXI	5345–6898	1554	5347–6900	1554	GTG	TAA	**+**
tRNA-Ser	6889–6960	72	6891–6961	71			**-**
tRNA-Asp	6961–7029	69	6962–7030	69			**+**
COXII	7033–7722	690	7034–7723	690	ATG	TAA	**+**
tRNA-Lys	7731–7797	67	7732–7798	67			**+**
ATP8	7799–7966	168	7800–7967	168	ATG	TAG	**+**
ATP6	7957–8670	714	7958–8671	714	ATG	AGA	**+**
COXIII	8636–9421	786	8637–9422	786	ATG	TAA	**+**
tRNA-Gly	9430–9498	69	9431–9499	69			**+**
ND3	9500–9850	351	9501–9851	351	ATG	TAA	**+**
tRNA-Arg	9857–9922	66	9858–9923	66			**+**
ND4L	9931–10,221	291	9932–10,222	291	ATG	TAA	**+**
ND4	10,215–11,591	1377	10,216–11,592	1377	ATG	TGA	**+**
tRNA-His	11,592–11,660	69	11,593–11,661	69			**+**
tRNA-Ser	11,661–11,728	68	11,662–11,729	68			**+**
tRNA-Leu	11,730–11,801	72	11,731–11,802	72			**+**
ND5	11,803–13,599	1797	11,804–13,600	1797	ATG	AGA	**+**
ND6	13,570–14,103	534	13,571–14,104	534	ATG	TAG	-
D-loop1	14,104–15,049	946	14,105–15,131	1027			**+**
tRNA-Thr	15,050–15,121	72	15,132–15,203	72			**+**
tRNA-Glu	15,185–15,255	71	15,569–15,639	71			-
D-loop2	15,256–15,489	234	15,640–15,849	210			**+**
CYTB	15,490–16,680	1191	15,850–17,040	1191	ATG	AGA	**+**
tRNA-Pro	16,683–16,753	71	17,043–17,113	71			-

Moreover, both species have two D-loop regions with rearrangement between ND6 and CYTB (Table 3). The D-loop1 and D-loop2 sizes for *C*. *hellenicus* were 946 and 234 bps, respectively. The sizes for *C*. *graecus* were 1,027 and 210 bps. Interestingly, a repeat of 39 bp (ATGTAATTACATAGGTATATGCCTCTATGGCATAGGTAT) was detected in D-loop1; it was found six and eight times on *C*. *hellenicus* and *C*. *graecus*, respectively. A different type of repeat was recorded between the tRNA-Thr and tRNA-Glu. This repeat originated at the 3′-end of the tRNA-Thr and the complementary 3′-end of the tRNA-Glu [36]. The region was found once on *C*. *hellenicus* and was repeated six times on *C*. *graecus*. Finally, the D-loop2 of *C*. *hellenicus* had six repetitive sequences (two different arrays), whereas *C*. *graecus* had five repeats (one array; AATTGTAATTTTAAAATTTCTTTTTT, 26 bp).

### Phylogeny and divergence within Petromyzontiformes

**Fig. 3:**
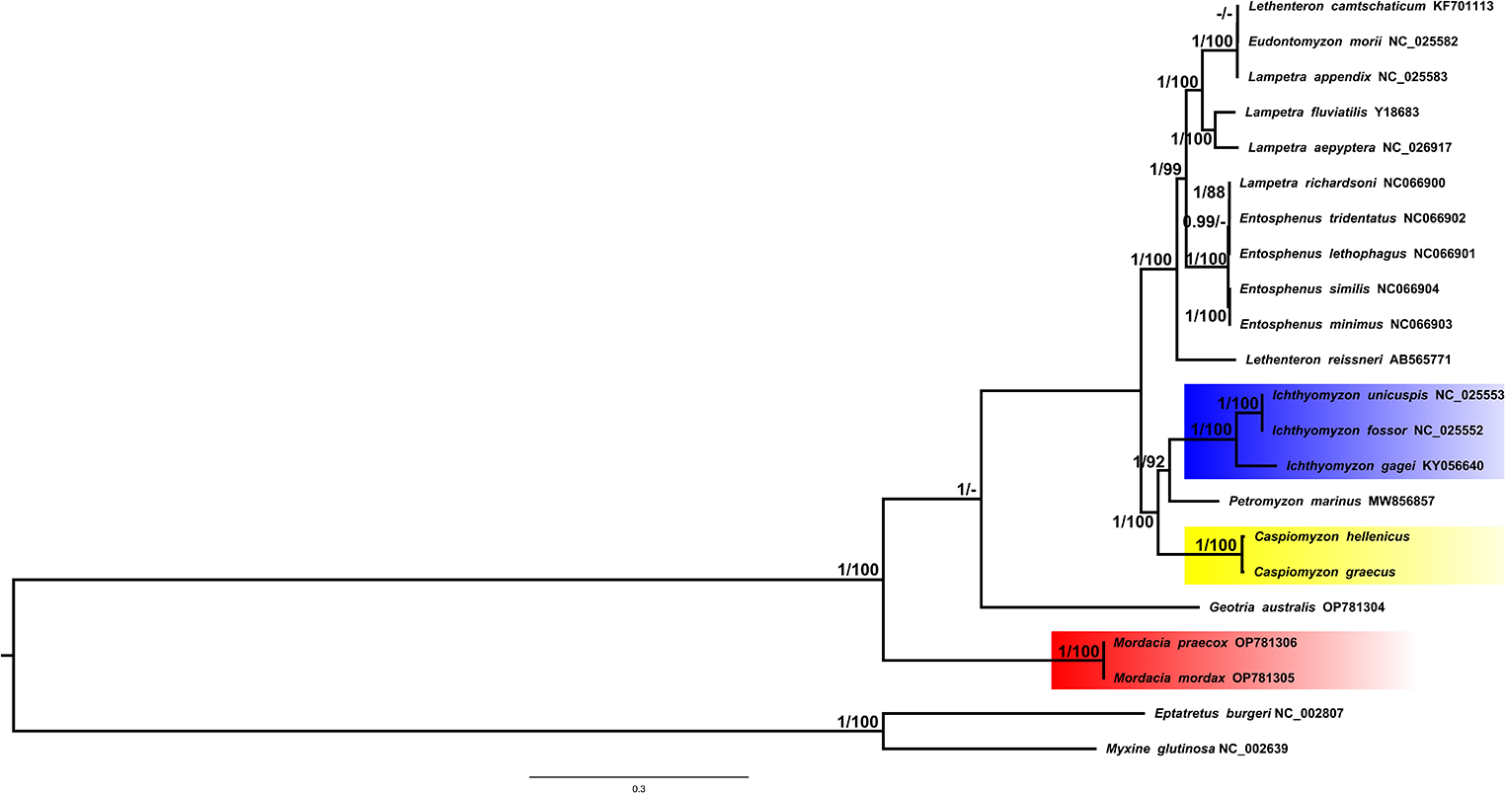
Bayesian phylogeny of extant lamprey species inferred from 13 protein-coding genes (PCGs), and two ribosomal RNA genes (12S and 16S ribosomal RNA). The tree is rooted with *Eptatretus burgeri* and *Myxine glutinosa*. Values in branches indicate support for each node based on Bayesian/ML inference. Bootstrap values under 80% are not shown.

**Fig. 4:**
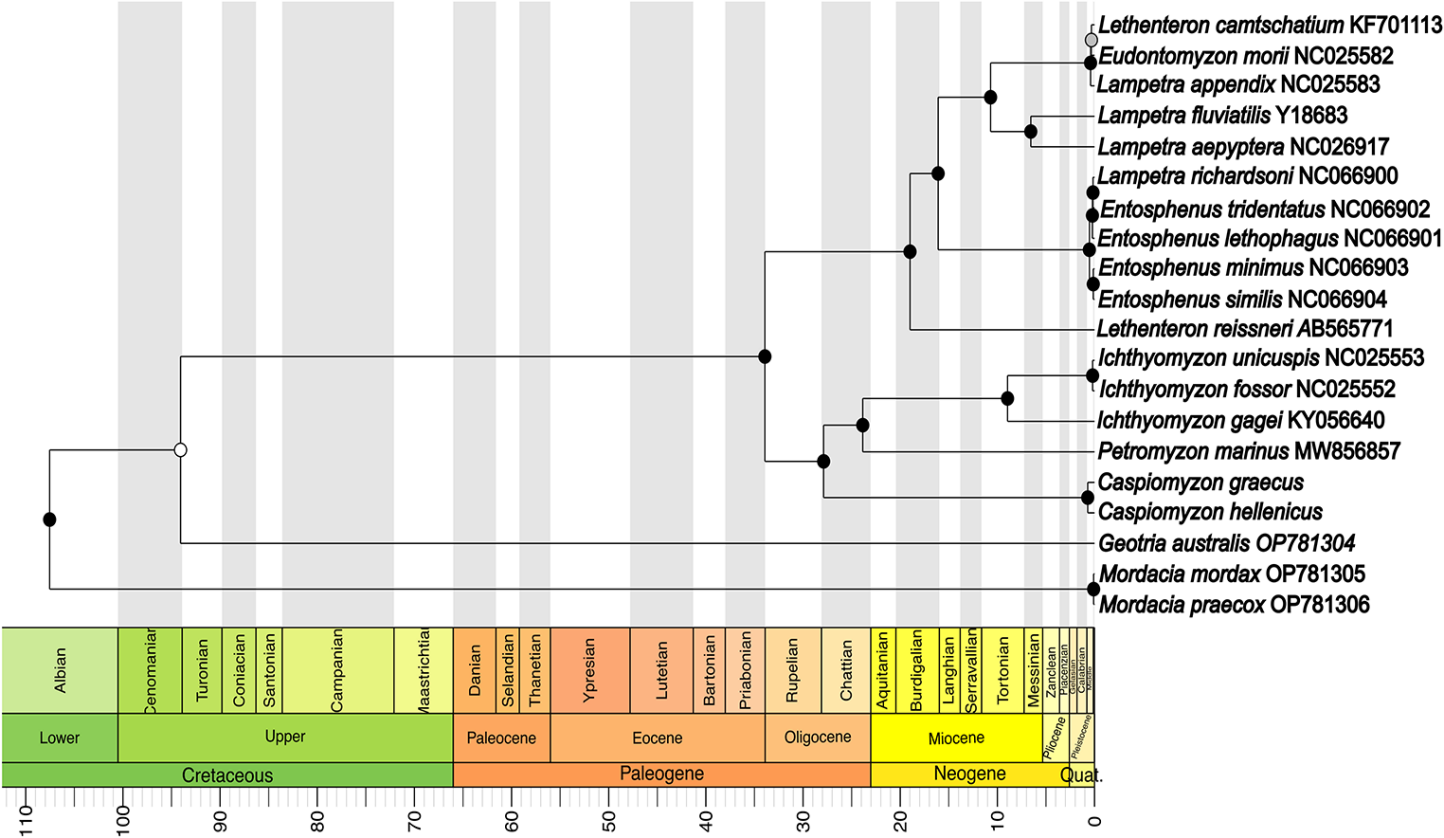
Tip-dated phylogenetic tree of crown-group lampreys inferred from two independent runs using BEAST 2.7.7.

The phylogenetic topologies inferred by both methods (ML and BI) were identical with well-supported values (Fig. 3). Both trees recovered three main clades, separating the species that belonged to the Petromyzontidae, Geotriidae, and Mordaciidae families. The single *Geotria australis* was sister to all northern hemisphere lampreys, whereas the two *Mordacia* species formed a monophyletic group that nested outside the other species. Two clades were also identified within the Petromyzontidae family; the first comprised of both Greek species (*C*. *hellenicus* and *C*. *graecus*) and their sister group *Petromyzon* and *Ichthyomyzon*. Moreover, *Lethenteron* and *Lampetra* were not monophyletic within the second clade, as they both appeared in two and three subclades, respectively. Interestingly, *Lampetra richardsoni*, the only species from the genus found in the western Nearctic, is grouped with species of the same zoogeographic realm and the genus *Entosphenus*. The calibrated species tree (Fig. 4) separated the northern lampreys from Geotriidae during late Cretaceus at approximately 94 Mya (confidence interval CI 118.63–70.38 Mya). The *P*. *marinus*, *Ichthyomyzon*, and *Caspiomyzon* group separated from the *Eudontomyzon*, *Entosphenus*, *Lethenteron*, and *Lampetra* species around 33.92 Mya. Divergence between the Greek *Caspiomyzon* species took place during late Pleistocene, at approximately 0.7 Mya (CI 1.05–0.39 Mya).

## Discussion

### Mitogenome characterization and annotation

The mitochondrial genomic organisation of both *Caspiomyzon* species was identical to that reported from other Petromyzontiformes [37–38]. Only one coding gene (*nd6*) and eight tRNAs occurred in the negative strand, whereas the remaining PCGs, tRNAs, and rRNAs were on the positive strand (Table 3), a pattern similar to all currently available mitochondrial genomes of Petromyzontidae (see Table 2 for available mitochondrial genomes). Additionally, lamprey mitochondrial genomic rearrangements were recorded close to the non-coding genes, similarly to invertebrate (e.g., sea urchins) and other vertebrate (e.g., loaches, carps) mitochondrial genomes [37]. The mitogenome length of both Greek species was the largest behind the mitogenomes of *Mordacia* and *Geotria* species (Table 2). This was mainly due to the large size of both non-coding regions (D-loop1 and D-loop2), and the occasional insertions and/or deletions in tRNA and rRNA genes [39]. The overall length of the other genes is highly similar to that of other Petromyzontiformes [37–38].

The repetitive sequences in lamprey mitochondrial DNA control regions are highly variable, both in copy number and in nucleotide composition [36]. The copy number variation in both control regions, as well as those emerging from tRNA genes, is probably attributed to slipped-strand mispairing [36, 40]. We detected two different repetitive arrays on the D-loop2 of *C*. *hellenicus*, whereas only one array was found on *C*. *graecus*. However, we sequenced only one specimen per species and could not corroborate copy variability within species. Such variability has been recorded within populations of various lamprey species, invalidating the D-loop from been an ideal genetic marker for species identification [36]. Interestingly, we also detected a novel repeat that was present between the tRNA-Thr and tRNA-Glu in both species, with slipped-strand mispairing as the most plausible explanation [36]. The characterization of this repeat was first described on other non-parasitic species of the genus *Lethenteron* [36], with an entire freshwater life cycle similar to both *Caspiomyzon* species. The repeat was not detected in parasitic species; therefore, a detailed analysis on the presence of such repeats on other non-parasitic species would be beneficial to comprehend the evolutionary history of lampreys.

### Petromyzontiformes phylogeny and divergence

The classification and phylogenetic relationship among lamprey species have always been controversial [14, 19, 41]. Our results corroborate the taxonomy of the group from some studies [19, 41–42], confirming the paraphyly of the southern hemisphere families. Mordaciidae was separated from the second southern hemisphere family (Geotriidae) and the northern Petromyzontidae (Figs. 3 and 4). The latter families were sister groups, contradicting the previous separation of southern families from the single north hemisphere family [14, 43]. Additionally, the lack of monophyly of both *Lethenteron* and *Lampetra* and the problematic recovery of some clades was confirmed [19, 39]. Attempts with different outgroups using mitochondrial markers had similar results [19]. The systematics and evolutionary history of extant lamprey species have been continuously updated with the inclusion of additional markers, the taxonomic revision of existing species [14, 16, 43], and the discovery of new species in both hemispheres [42]. Previous studies have used a few molecular, mainly mitochondrial cytochrome oxidase subunit I and cytochrome b, and/or morphological markers to examine the phylogeny and the historical biogeography of lampreys [16, 19, 43], whereas almost half lamprey species were not included in our phylogenetic analyses. High-throughput data have been recently used to delineate lamprey phylogeny confirming the monophyly of northern and southern Hemisphere species, however, it did not include all extant species [14].

The time-calibrated phylogeny showed that the separation of the three lamprey families occurred during the late Cretaceous, corroborating recent estimates from protein-coding regions of various genes [14]. Additionally, some extant species in both hemispheres and their lineages exhibit very recent divergence (Fig. 4). The Petromyzontidae family was formed around 94 Mya following the consecutive breakups of Pangea. Interestingly, the genus *Caspiomyzon* was separated from the marine *Petromyzon marinus* at late Paleogene (~ 28 Mya), just after the formation of Paratethys and the creation of the various mountains in Europe and parts of western Asia. Differences in age diversification estimates among studies have been recorded [14, 16, 43]. The inclusion or not of fossil records, outgroup species, and taxonomic sampling could have potentially affected the results.

Among the species exhibiting speciation during the late Quaternary, the Greek lampreys show the oldest records (0.7 Mya). Greece includes five out of the eight biogeographical regions in the Balkan peninsula [7] that were mainly shaped by major geological events [44]. The latter created obstacles to species dispersal that could have consequently accelerated vicariance speciation. Each Greek lamprey species is found in different regions with distinct evolutionary histories; *C. hellenicus* is located at the Aegean region (Thracian subregion), whereas *C. graecus* is found in the Ionian region. Moreover, Quaternary glaciation cycles have largely shaped the evolution and distribution of freshwater species [45]. These events have been suggested to drive diversification among other freshwater taxa in the area [46]. However, morphological similarities and limited differences across complete mitogenomes of congeneric lampreys could also be indicative of recent ecotypes rather than speciation in lampreys [47]. Our results highlight that additional markers (e.g., whole genome data) could effectively provide a higher phylogenetic resolution for lampreys and other non-model species [14, 48].

## Conclusion

In this study, we annotated the complete mitochondrial genomes of two Greek lampreys with ONT and we used available data to understand the systematics of the species (Table 3). Both Greek lampreys exhibit a very restricted distribution which are under serious threats [Sapounidis et al., submitted; Xanthopoulou et al., submitted]. They are also found in regions that have different evolutionary history as seen in other freshwater fishes. *Caspiomyzon hellenicus* original habitat at Tenagi is currently transformed into an intensive agricultural landscape, limiting its presence to two small water bodies (Aggitis River and Kefalari springs). Moreover, *C. graecus* has an even more constricted distribution (Filippias tributary) in Epirus, Northwest Greece [17]. Additionally, the species were recently taxonomically revised and assigned to the genus *Caspiomyzon* from *Eudontomyzon* [19, 43]. However, these studies have used a single molecular marker (cytochrome b sequences); therefore, the phylogenetic and evolutionary reconstruction of the Greek lampreys with a robust molecular dataset are imperative. More work on Greek lampreys is required as no targeted biological assessments on the species has ever been undertaken. The emerging threats to freshwater biodiversity and ecosystems include pollution, habitat destruction, overfishing, invasive species, and escalating climate change. Such pressures could affect the hydrological characteristics and geomorphological features of the landscape and have significantly affected the abundance and distribution of both endemic and threatened species [Sapounidis et al., submitted; Xanthopoulou et al., submitted]. Currently, the ecological water quality of Tenagi has been assessed for the first time using the Fish-based River Integrity Index [49]. Results showed that the water quality in almost all stations is “moderate”, “poor” in two, and only one station had an overall “good” index [Sapounidis et al., submitted]. Similar efforts should focus on freshwater ecosystems that host other lampreys in Greece (Louros river), particularly in areas where there are indications of undescribed species (e.g., *Eudontomyzon* sp. Almopaios; Almopeos River) [5].

## Data Availability

Data are deposited on GenBank with accession numbers: PQ845996-PQ845995.
